# Nutritional Properties of Dietary Omega-3-Enriched Phospholipids

**DOI:** 10.1155/2013/965417

**Published:** 2013-07-31

**Authors:** Elisabetta Murru, Sebastiano Banni, Gianfranca Carta

**Affiliations:** Università degli Studi di Cagliari, Dipartimento di Scienze Biomediche, Cittadella Universitaria, S.S. 554, km. 4,500, Monserrato, 09042 Cagliari, Italy

## Abstract

Dietary fatty acids regulate several physiological functions. However, to exert their properties, they have to be present in the diet in an optimal balance. Particular attention has been focused on tissue highly polyunsaturated fatty acids (HPUFAs) n-6/n-3 ratio, influenced by the type and the esterified form of dietary fatty acids. Dietary EPA and DHA when esterified to phospholipids (PLs) are more efficiently incorporated into tissue PLs and seem to possess peculiar properties through specific mechanism(s) of action, such as the capacity to affect endocannabinoid biosynthesis at much lower doses than EPA and DHA in triglyceride form, probably because of the above mentioned higher incorporation into tissue PLs. Downregulation of the endocannabinoid system seems to mediate the positive effects exerted by omega-3-enriched PLs on several parameters of metabolic syndrome. PLs are one of the major dietary forms of EPA and DHA we are exposed to with the everyday diet; therefore, it is not surprising that it guarantees an effective EPA and DHA nutritional activity. Future studies should address whether EPA and DHA in PL form are also more effective than other formulations in ameliorating other pathological conditions where n-3 HPUFAs seem to exert beneficial activities such as cancer and psychiatric disorders.

## 1. Introduction

Dietary fatty acids have a wide range of physiological functions, and, to fully exert their effects, they have to be present in the diet in a certain balance. Thus a proper amount of all families of fatty acids needs to be taken into account in making dietary recommendations. Particular attention has been paid to the ratio between highly polyunsaturated fatty acids (HPUFAs) n-6 (mainly arachidonic acid (20 : 4n-6; ARA)) and n-3 HPUFAs (mainly EPA and DHA) in tissues as a consequence of dietary n-6/n-3 ratio, since an inverse correlation between circulating n-3 HPUFAs and the occurrence of coronary heart disease has been observed [1]. 

In addition, dietary n-3 HPUFAs have been shown to prevent and modulate essential fatty acid (EFA) deficiency in infancy (retinal and brain development), autoimmune disorders, Crohn's disease, and cancers of breast, colon, and prostate. They have also been shown to exert beneficial effects in a wide range of psychiatric disorders [2]. However, even though they appear as the panacea for any pathological states, some epidemiological studies cast doubts on some of these beneficial effects [3]. It is therefore compelling to individuate the mechanism(s) of action and what are the conditions where n-3 HPUFAs maximise their nutritional activities. The metabolism and biological activities of marine n-3 PLs have been recently reviewed [4]; in the present paper, we therefore focused our attention on the putative mechanisms of action by which EPA and DHA in PL form exert their distinct effects with respect to the triacylglycerol (TAG) form.

## 2. Effects of EPA and DHA on Oxygenated Eicosanoid Biosynthesis 

It has been shown that EPA and DHA, besides possessing antithrombotic properties [5], are able to decrease both the formation and tissue incorporation of ARA and, hence, to reduce the release of inflammatory acute-phase proteins [6, 7]. 

In fact, proinflammatory eicosanoids, including prostaglandin E_2_ (PGE_2_) and leukotriene B_4_ (LTB_4_), are synthesized from ARA on phospholipase-mediated release from cellular phospholipids (PL) [8–10], while n-3 HPUFAs are prostaglandin and leukotriene precursors (PGE_3_ and LTB_5_), with a quite low conversion coefficient, with anti-inflammatory properties [11–13]. 

The daily endogenous formation of PGs is much lower than the daily ARA intake and the endogenous ARA production. EPA and DHA inhibit the in vitro production of ARA-derived eicosanoid [14, 15], but not in vivo, where eicosanoid formation seems to be less affected by EPA and DHA dietary intake [16, 17]. To observe a slight decrease of in vivo production of ARA-derived eicosanoids several grams per day of EPA and DHA for many weeks are needed therefore the beneficial effect of a relatively low dose of n-3 HPUFAs on death from coronary heart disease in the GISSI study [18] is unlikely to be associated with changes in eicosanoid production. In vivo PG production by dietary supplements with pure ARA (e.g., 6 g/day for 2-3 weeks in humans) can be slightly increased [19]. These studies suggest that eicosanoid formation is not promptly influenced by ARA dietary intake, at least within the range of traditional human diets [20, 21]. However, it has been shown that very high amounts of fish oil may decrease PG formation with consequent weak anti-inflammatory and analgesic effects [22].

Prescott showed that isolated human polymorphonuclear leukocyte neutrophil (PMNs) incorporated EPA, mainly in phospholipids (PLs), and were able to convert it to LTB5, suggesting an impairing replacement of ARA with EPA in PMN membranes [23]. These results are in contrast with the report of Lammi-Keefe and coworkers, who did not observe such an effect on neutrophils probably because of the low amount of EPA consumed with the dietary supplement in their experiments [24]. 

Therefore, these mechanisms may only explain in part the effects demonstrated in different experimental conditions [25–27].

## 3. Interaction with Other Dietary Fatty Acids

EPA + DHA supplementation, with or without different levels of gamma-linolenic acid (GLA, 18 : 3n-6), was shown to induce changes on fatty acid profile [28]. Intake of both GLA and EPA + DHA decreased ARA concentrations in animal tissues and cells [29, 30], not in humans supplemented with GLA + EPA (1 : 1) [31]. However a significant reduction in ARA concentrations was observed in serum PLs, likely induced by the competition with EPA and dihomo-*γ*-linolenic acid (DGLA) for esterification into cellular PLs and the attenuating effect of n-3 fatty acids on Δ5-desaturase, necessary for the conversion of DGLA to ARA [32, 33].

## 4. Does Dietary Form of PUFAs Affect Their Lipid Incorporation and/or Nutritional Activities?

It is often overlooked what is the most efficient dietary form to convey dietary fatty acids in terms of tissue bioavailability and biological effects. The choice of the form is mostly dictated by practical or economic reasons. However, there are several reports showing that PL-bound EPA and DHA have distinct effects with respect to TAG-bound EPA and DHA.

Fish oil supplementation improves lipoprotein profiles by reducing plasma LDL, cholesterol, and TAG levels and increasing HDL cholesterol [34–36] through inhibition of TAG and VLDL synthesis in the liver [37, 38]. Such effects have been generally obtained through prolonged supplementation of 1 or more g/day of EPA and/or DHA, marketed either as TAG or ethyl ester (EE). In a 5-week supplementation of 4 g EPA or DHA, as EE, added to the ordinary diet, EPA showed a more rapid and comprehensive increase in serum PLs than did DHA [39].

Interestingly, it has been shown that EPA and DHA as krill powder are able to significantly decrease plasma triglycerides (TAGs) at much lower doses, about 400 mg per day [40].

However, it cannot be ruled out if some of the effects may be related to different actions of EPA and DHA. In fact some authors reported that EPA accumulated faster than did DHA in plasma [41, 42], leukocytes [43], and erythrocytes [44]. Willumsen et al. reported that EPA decreased serum TAGs in rats, probably through an induction of FA mitochondrial oxidation, whereas DHA did not [45]; however, other authors reported that DHA had similar TAG-lowering effect to EPA [39, 46]. Conversely others reported a concomitant increase of total, LDL, and HDL cholesterol concentrations when n-3 HPUFAs, particularly DHA, have been administered in amounts of at least 1 g/day [47]. Therefore, when comparing the effects of the form, a similar EPA/DHA ratio should always be used [48].

In addition, it has also been suggested that the food matrices may affect the bioavailability. Visioli and coworkers observed that the administration of as low as 300 mg/day of EPA + DHA to healthy subjects, incorporated into milk, subdivided into smaller doses throughout the day, for 3–6 weeks, resulted in a significant increase in EPA and DHA levels in plasma lipids as well as in HDL concentrations, while TAG concentrations decreased without affecting total cholesterol concentration [49]. This is noteworthy since the same effects on TAG and HDL concentrations have been obtained with 1 to 7 g/day of EPA and DHA [50]. 

A recent paper by Galli et al. suggests that variable blood levels of n-3 HPUFAs are due to their presence as common dietary components and, possibly, are responsible, among other reasons, for the variability of the results observed; hence, it is recommended to apply a selection of subjects with uniform n-3 background levels before a trial begins [51]. 

Thus, it is quite difficult to make any recommendation about n-3 HPUFA doses since the matrices, the form, and the quality may greatly influence tissue bioavailability.

American Heart Association, AHA, dietary guidelines for n-3 HPUFAs and fish for primary prevention of coronary diseases, on a food-based approach [52], are two servings of fatty fish per week [53], 250–500 mg EPA + DHA per day [54], while it is suggested a larger daily intake, 1 g or more, for a TAG-lowering effect [55–57]. Indeed fish oils are major sources of n-3 HPUFAs, while other types of oil, especially vegetable oils, are a good source of ALA [58]. However, it should be borne in mind that even though ALA shares with EPA and DHA the double bond at the third carbon from the methyl end that characterises the n-3 PUFA family, and it is their dietary precursor, possesses distinct metabolism and nutritional properties [59].

## 5. Digestion, Absorption, and Bioavailability of PL-Bound EPA and DHA

Dietary fat is mainly composed of TAG with a small portion of PLs (3–6%) [60]. The daily intake of PL/day is 2–8 grams. The most common PL in the intestinal lumen is phosphatidylcholine (PC) which is derived mostly from bile (10–20 g/day in humans) and also from the diet, while other PLs, such as phosphatidylethanolamine (PE), phosphatidylserine (PS), and phosphatidylinositol (PI), are present in much smaller amounts. 

Fatty acid chain length and unsaturation number influence fat digestion, absorption, transport in the blood, and metabolism at cellular level. Medium-chain fatty acids (MCFA) are better absorbed than longer fatty acids because they can be dissolved in the aqueous phase and then be absorbed bound to albumin and transported to the liver directly by the portal vein [61]. Dietary TAGs structure influences the bioavailability of fatty acids. For example palmitic acid in fat human milk is well absorbed because of its incorporation in the sn-2 position of glycerol backbone [62]. On the other hand, whether fatty acids esterified in TAGs or in PLs impact on their absorption and tissue incorporation is still debated. TAGs and PLs are digested and absorbed in different ways in the small intestine. The first requires emulsification by bile salts while PLs can spontaneously form micelles and be conveyed in an aqueous environment. According to studies in humans and animals, oral administration of labeled PC leads to the rapid appearance of labeled PC in plasma, with rather weak formation of labeled TAGs [63]. In contrast to TAGs, PLs are not hydrolyzed by lingual or gastric lipases but only in the small intestine [64]. After oral administration, PC is more than 90% absorbed by the intestinal mucosa via conversion to LysoPC because PC is hydrolyzed by pancreatic phospholipase A2 (PLA2) which releases the fatty acid from the sn-2 position. After absorption by the enterocytes, this LysoPC can be reacylated into PC, while the previously released fatty acid can be used for TAG synthesis [65–67]. The absorbed PC is then incorporated into chylomicrons (CM) and, after degradation to the TAG-rich particles, is taken up by the high-density lipoprotein (HDL) fraction which occurs relatively rapidly, that is, within 5-6 hours of PL ingestion [66, 68]. A small proportion of this PC is taken up without prior hydrolysis. Therefore, dietary PLs may affect the lipoprotein composition and metabolism. It has been reported that lipoproteins secreted by the rats small intestine after the infusion of triolein were CM, whereas those secreted after egg PL infusion were VLDL-size particles [69]. Recently, piglets fed with HPUFA-TAG formula had higher HPUFA content in LDL phospholipids than those fed with HPUFA-PL formula. The opposite results were found in HDL PLs, indicating that dietary HPUFAs in form of TAG or PL differently affect the composition of HDL and LDL PLs [70]. Therefore, PLs in the lipoproteins can influence the distribution of lipoproteins in the body and fatty acid tissue incorporation.

Some studies in newborn infants have indicated that dietary PLs may be better absorbed than TAGs. In particular, a study on a group of preterm infants fed with different formulae has shown that the absorption of DHA was higher in those infants receiving the HPUFA-PL formula than in infants receiving breast milk or the HPUFA-TAG formula [71]. 

Maki et al. showed that EPA and DHA from krill oil (KO), mainly bound to PLs, were absorbed at least as efficiently as EPA and DHA from fish oil (FO), which are in the TAG form [72]. However, Schuchardt et al. [73] compared the uptake of three EPA + DHA formulations derived from fish oil (reesterified (rTAG), EE, and KO, mainly PL) and showed that the bioavailability of n-3 HPUFAs may vary according to their esterified form. The highest incorporation of EPA + DHA into plasma PLs was obtained by KO, followed by FO rTAG and then by EE. A study on the incorporation of labeled DHA into platelet and red cell PLs showed that, in platelets, [13C]DHA accumulated in both PC and PE, although a little faster in PC [74]. Also the availability of LysoPC and LysoPE has been well documented in platelets [75–77]. In contrast to platelets, [13C]LPC could be detected in erythrocytes when [13C]DHA started rising in PC [78]. This is in agreement with the hypothesis that DHA is preferentially esterified to LPC in erythrocytes [79] and is subsequently reacylated into PC [80]. Interestingly, DHA levels in erythrocytes may be considered, at least in part, as an index of that present in the brain, and LysoPC might be a preferential vehicle of DHA to the brain, as suggested in young rats [81, 82]. 

## 6. Biological Activities of PL-Bound EPA and DHA

n-3 HPUFAs in fish products are mainly bound to PLs, while in fatty fish, such as salmon, n-3 HPUFAs are bound to PLs and TAGs in a 40 : 60 ratio [83]. On the contrary, most of n-3 HPUFAs available as supplement, like FO, consist almost exclusively of TAG-bound n-3 HPUFAs. This seems like an important issue since dietary PLs have consistently been shown, per se, to affect plasma and liver lipid levels in experimental animals [84]. A recent work has demonstrated that the addition of dairy milk PLs to the diet at a level of 2.5% by weight (wt%) can reduce cholesterol and TAG levels in high-fat-fed C57BL/6 mice [85].

KO, extracted from Antarctic krill (*Euphavsia superba*) [86], is a relatively new source rich in n-3 HPUFAs in the form of PLs, mainly PC, rather than TAGs; the proportion of PLs in the total lipids of krill has been reported to vary between 30 and 60%, depending on krill species, age, season, and harvest time [87]. Moreover, KO contains a lipid-soluble antioxidant, astaxanthin, that may preserve KO from oxidation. The peculiar composition may influence the tissue bioavailability of n-3 HPUFAs and might be responsible, in part, for the health-promoting effects of KO, such as its anti-inflammatory and hypolipidemic properties in humans [88, 89].

A daily intake of 3 g KO, containing 543 mg EPA + DHA, increased the plasma level of EPA and DHA to the same extent as dietary FO, containing 864 mg EPA + DHA [90], suggesting a comparable absorption for EPA and DHA after KO treatment, if not better than after FO treatment. There was, however, no significant difference across the treatments (KO, FO, and control) for TAG and lipoprotein response, confirming a previous study that also failed to detect any change in plasma lipids [91], probably because of the short period of treatment, the normolipidemic status of the subjects, or the low amount of EPA and DHA. On the other hand, in another study, a small increase in LDL cholesterol and no effect on HDL cholesterol were found after FO treatment [92]. Another controversial aspect is the hs-CRP, a marker of systemic inflammation, which showed no change among KO, FO, or placebo treatments [91, 93, 94], while in individuals with a proinflammatory status, an association among DHA/EPA and hs-CRP reduction has been reported [95]. 

In mice fed with high-fat diet, it has been demonstrated that n-3 HPUFA in PLs is more efficacious than TAG in reducing hepatic steatosis, low-grade inflammation in white adipose tissue [96], blood lipid levels, and glycaemia [97, 98]. Moreover, increased hepatic expression of fatty acid oxidation genes and downregulation of lipogenic genes were observed. The latter effect was stronger in the PL-treated mice, and a reduced plasma insulin and adipocyte hypertrophy was observed only with the PLs form [96]. It is likely that supplementation of n-3 HPUFA as PLs exerts stronger biological effects compared with the TAG form because (i) various PL species can also act as ligands for nuclear receptors involved in the transcriptional regulation of steroidogenesis and cholesterol metabolism [99, 100] and (ii) the PLs form has been shown to augment the bioavailability of DHA and EPA in both rodents [69, 101] and humans [71, 89, 102]. 

Other areas where EPA and DHA have been claimed to have an influence are neuropsychiatric disorders. Results from several epidemiologic studies [103, 104] suggest that dietary consumption of n-3 HPUFAs affects neuropsychiatric disorders, presumably because of their structural and neurochemical involvement in pathophysiological processes [105–107]. Interventions ranging from 1 to 6.2 g/d EPA and from 0 to 3.4 g/d of DHA were associated with a therapeutic effect in a broad spectrum of psychiatric disorders [108–111]. Children supplemented with n-3 HPUFA PLs and FO showed an increase in EPA, docosapentaenoic acid (DPA), and DHA concentrations and a decrease in ARA and adrenic acid concentrations in plasma PLs but not in TAGs and cholesterol ester fractions [112], as observed in previous studies [113]. A negative feedback mechanism on desaturase enzymes [114, 115] could be associated with these alterations. On the other hand, the same study showed limited effects on n-3 HPUFA concentrations and none on n-6 HPUFA concentrations in erythrocytes following n-3 HPUFA PLs and FO supplementation [112]; these observations are inconsistent with a previous study [116]. The controversial findings could be explained by the low n-3 HPUFA dose intervention and the kinetics of dietary EPA and DHA and/or the participants' metabolism. 

In Vaisman study total test of variables of attention (TOVA) score increased in patients supplemented with n-3 HPUFA PLs and with FO with a limited extent, providing about 250 mg/d EPA/DHA. These findings differ from previous reports in which even larger amounts of DHA [117] or EPA + DHA [118] supplementation in attention-deficit hyperactivity disorder (ADHD) children for 2–4 months were shown to result in elevated plasma PLs DHA concentrations and in no effect on a continuous performance test (CPT) scores. 

Although dietary manipulation of n-3 HPUFAs in the brain is complicated by the high concentrations in this organ, supplementation of DHA-containing PLs, such as the bovine brain cortex PS, to animal models was shown to attenuate neuronal effects of aging [119] and to affect behaviour as well [120, 121]. DHA supplementation of 345 mg/d or 3.6 g/body wt, in ADHD children, provided as EE [117] in capsules or as TAG in functional food [118], induced a pronounced increase in blood n-3 HPUFAs, but failed to affect ADHD symptoms. Results of recent studies, in which EPA- and DHA-enriched FOs were provided along with n-6 HPUFA-containing oils and/or vitamins to ADHD children, were conflicting [116, 122–124]. Interestingly, early observations suggested that dietary HPUFAs esterified to PLs rather than TAGs are more effective substrates for brain tissues accretion in term baboons [125].

## 7. PL-Bound EPA and DHA May Exert Their Biological Activity by Affecting Endocannabinoid Biosynthesis 

Different studies support the hypothesis that the composition of dietary fatty acids can affect energy homeostasis via changes of endocannabinoid system (ECS). The endogenous cannabinoid system is an ubiquitous lipid signaling system that appeared early in evolution and which has important regulatory functions throughout the body in all vertebrates. The main endocannabinoids are molecules derived from ARA hydrolyzed from membrane PLs. In particular, anandamide (arachidonoylethanolamide or AEA) [126–128] derives from hydrolysis of membrane PLs at the sn-1 position, while 2-arachidonoylglycerol (2-AG) derives from hydrolysis at the sn-2 position [129–132]. They bind to a family of G-protein-coupled receptors, of which the cannabinoid CB1 receptor is widely distributed in different brain areas and peripheral tissues. However, by the same biosynthetic pathway of anandamide, other structure-related lipid messengers, palmitoylethanolamide (PEA) or oleoylethanolamide (OEA), are formed by palmitic acid or oleic acid, respectively, esterified in sn-1. Being the latter fatty acids preferentially incorporated in sn-1, PEA, and OEA are more abundant than anandamide. They exert their biological activity by interacting with other receptors. OEA, an anorectic mediator that affects lipid and glucose metabolism, activates PPAR-alpha [133]. PEA exerts anti-inflammatory actions via direct activation of PPAR-alpha [134], transient receptor potential of vanilloid type-1 (TRPV1), or PPAR-gamma [135]. 

From different studies it is reasonable to hypothesize that the tissue levels of endocannabinoids are in part regulated by the activity of the corresponding biosynthetic precursors and in part by catabolic enzymes, fatty acid amide hydrolase (FAAH) [136, 137], or monoacylglycerol lipase (MAGL) [138, 139]. ARA and possibly also glycerol and ethanolamine, produced from the hydrolysis of 2-AG and AEA, are rapidly incorporated into membrane PLs [140, 141]. The tissue levels of endocannabinoids may also depend on the availability of their biosynthetic precursors ARA in PLs [142–144]. In fact, the diet-induced changes were accompanied by changes in the corresponding fatty acids esterified to individual PLs. Watanabe et al. [145] found that mice fed with an n-3-PUFA-deficient diet exhibited higher brain 2-AG levels. Furthermore, short-term supplementation of DHA-rich FO reduced brain 2-AG levels as compared with the diet supplemented with low n-3 PUFAs. The authors observed a concomitant decrease in ARA levels and an increase in DHA levels in the major brain phospholipid species of mice fed with the FO diet as compared with those fed with the low n-3 PUFA diet. 

The concentrations of endocannabinoids and their congeners could be modified by the dietary content of HPUFAs or by their essential biosynthetic precursors, mainly in peripheral tissues. A study has been carried out in vitro to determine whether incubation of cells with certain free fatty acids can affect locally produced AEA and 2-AG levels [146]. It has been shown that incubation of 3T3F442A mouse adipocytes with ARA strongly elevates 2-AG levels as well as the amounts of ARA esterified in TAGs and on the glycerol sn-2 position, but not on the sn-1, into PLs, whilst incubation with DHA decreased 2-AG and AEA levels and the amounts of ARA esterified on both sn-2 and sn-1 positions of PLs, but not on TAGs. This suggests that dietary HPUFAs might modulate fatty acid composition of adipocyte PLs that act as endocannabinoid precursors, and then it can be assumed that n-3 HPUFAs might have their beneficial effects in abdominal obesity, dyslipidemia, and insulin resistance by CB1-mediated lipogenic actions of endocannabinoids in adipocytes [147–149]. It has been shown that endocannabinoids can affect energy metabolism both by stimulating food intake and by affecting energy processing in the adipose tissue, liver, pancreas, and skeletal muscle [147, 149, 150]. Indeed, it has been shown that increase of peripheral endocannabinoid levels in both fasted and postprandial obese and overweight individuals correlates with intraabdominal obesity, glucose intolerance, dyslipidemia, and dyslipoproteinemia [150–154]. Therefore, dietary fatty acids by modulating ARA levels in tissue PLs may influence endocannabinoid biosynthesis and thereby downregulate an overactive endocannabinoid system.

In a recent study [49], the effects of dietary n-3 HPUFAs, in the form of either FO or KO, balanced for EPA and DHA content, on liver and heart fat and inflammation in Zucker rats (a model of obesity and related metabolic dysfunctions), were analyzed, as well amount of AEA and 2-AG in the abdominal and subcutaneous fat, liver, and heart. Rats fed with n-3 HPUFA diets had significantly lower liver TAGs and reduced peritoneal macrophage response to inflammatory stimulus than control rats; only in KO-fed rats, heart TAGs were significantly lowered. These effects were associated with a lower concentration of the endocannabinoids, AEA and 2-AG, in the visceral, but not subcutaneous, adipose tissue, and of AEA in the liver and heart; these decreased endocannabinoid levels were, in turn, associated with lower levels of ARA in membrane PLs. Since also CB1 antagonists can produce anti-inflammatory effects in macrophages [155], the observed n-3 HPUFA-induced reduction in endocannabinoid levels in the visceral adipose tissue might also be responsible for the dampened inflammatory response caused by FO and KO. 

The dietary imbalance among macronutrients leads to metabolic derangement of glucose and lipid disposal characterized by a marked dyslipidemia, increased insulin resistance, and fatty liver, which are some of the characteristic features of the metabolic syndrome.

Two studies [49, 96] showed that dietary DHA and EPA in the form of PLs are superior to TAGs with respect to the preservation of glucose homeostasis and the reversal of hepatic steatosis, adipocyte hypertrophy, and low-grade inflammation. The higher efficacy of n-3 HPUFAs administered as PLs was associated with their better PL bioavailability and with a relatively strong suppression of the levels of major endocannabinoids in white adipose tissue and plasma, suggesting that modulation of the endocannabinoid system activity contributed to their greater efficacy when compared to TAG form of n-3 HPUFA. The pathophysiological role of the endocannabinoid system in the development of adipose tissue inflammation [146] or hepatic steatosis [147, 148] is well described; moreover, the anti-inflammatory effects of adipocyte-derived N-acyl ethanolamines EPEA or DHEA, that is, the amides of EPA and DHA, have recently been suggested to play a role [149, 156]. The increase in obesity prevalence in the United States may be associated with the increased consumption of linoleic acid (LA), the precursor of ARA, and hence the precursor of endocannabinoids [157, 158]. This hypothesis was supported by dietary experiments in mice fed with various diets differing in the LA content that was positively correlated with the levels of ARA, 2-AG, and AEA in PLs from liver and erythrocytes and also promoted accumulation of body fat [158]. On the other hand, addition of EPA and DHA to the diet resulted in a decrease of endocannabinoid levels in the liver and also in hypothalamus of mice fed with experimental diets with a high LA content [158]. The data further support the role of dietary LA as the key factor controlling the activity of the endocannabinoid system and the attenuation of this activity as the key mechanism underlying antiobesity effects of dietary n-3 HPUFA supplementation under these conditions. However, the role of various tissues in the metabolic impact of downregulated endocannabinoid system activity in response to n-3 HPUFA should be better characterized [159]. 

An association between elevated peripheral levels of endocannabinoids and the metabolic syndrome has been reported in a study of Piscitelli and coworkers [98]. They showed that 8 weeks of a high-fat diet increased endocannabinoid levels in all tissues except the liver and epididymal adipose tissue, while KO reduced AEA and/or 2-AG levels in all tissues but not in the liver, usually in a dose-dependent manner. KO was shown to affect levels of endocannabinoids in part by reducing the availability of their biosynthetic precursors. KO supplementation was also accompanied by elevation of PEA levels, and, given its role as anti-inflammatory agent [160] and the previous observation that n-3 HPUFAs exert a protective effect against muscle damage induced by the proinflammatory cytokine TNF-alpha [161], the authors speculate that increased PEA levels might protect skeletal muscle from the damaging effect of TNF-*α* and contribute, together with KO-induced elevation of adiponectin levels, to the anti-inflammatory effects of KO. Since PEA is dysregulated in several tissues of obese Zucker rats [162], these data might suggest that KO can potentially produce beneficial metabolic effects against dysmetabolism and inflammation in obesity also by reequilibrating the activity of PPARalpha.

## 8. Influence of PL-Bound EPA and DHA on the Brain Endocannabinoid System 

Various studies have explored the influence of dietary fatty acids on brain endocannabinoid concentrations, which, together with receptor CB1, are involved in the regulation of synaptic plasticity and other functions as the control of movement and sensory perceptions to mood and neurogenesis [163]. 

In piglets, milk formulations enriched in HPUFAs were able, after one month of administration, to significantly modify the levels of the corresponding N-acylethanolamines (NAEs) in various brain regions; in addition, feeding dietary ARA induced an increase in whole brain AEA levels in mice [164]. Of the two endocannabinoids, AEA seems to be the one most involved in adaptive mechanisms against stress and its consequences, while 2-AG is the one clearly involved in hyperphagia in animal models of obesity.

In mice brain, decreased levels of 2-AG, but not AEA, were induced by high dose of dietary n-3 HPUFA as FO, whereas the opposite effect was obtained with an n-3 HPUFA-deficient diet [145]. On the other hand, no changes have been observed in brain 2-AG and AEA concentrations of rats, to which EPA or DHA had been administered for one week [155].

Di Marzo et al. [165] observed that obese Zucker rats, fed for one month with low doses of n-3 HPUFA (0.5% of EPA + DHA, in the form of either FO or KO, equivalent to 0.8 en% in the rat diet and corresponding to 1.8 g/d in a 2000 kcal diet in humans), showed a rise in EPA and DHA levels in brain PLs of KO group, compared with FO group, and no changes in ARA levels. At the same time, levels of 2-AG in the brain were decreased in KO group but not in FO group, as previously observed by Watanabe et al. [145]. However, it is not clear if the increase of n-3 HPUFAs is sufficient to lower brain concentrations of 2-AG. Since these alterations were not associated with food efficiency and food intake, which are under the control of hypothalamus area, it was supposed that this area was not involved in such decreases or these were not sufficient to exert a significant effect on CB1 receptor activity in this brain area. Therefore, relatively low doses of KO to Zucker rats, which was previously shown to downregulate peripheral endocannabinoid levels, ameliorating some aspects of the metabolic syndrome [49], in the brain reduces only 2-AG levels, suggesting that the beneficial effect of KO on the metabolic syndrome is mostly exerted by modifying endocannabinoid levels in peripheral tissues. 

These data indicate that both the amount of dietary n-3 HPUFAs and the dietary form, as FO or KO, may influence EPA and DHA incorporation into brain lipids and consequently either the biosynthesis of ARA or its incorporation into PLs via its partial replacement with EPA and DHA [166]. Thus, even though most of the nutritional effects of n-3 HPUFAs may occur through the modulation of the levels of PL-derived metabolites, such as oxygenated eicosanoids [167], and of endocannabinoid concentrations [159], it remains to clarify on whether and how modulation of the biosynthesis of these bioactive compounds may influence brain activities.

## 9. Nutritional Activities of PL-Bound EPA and DHA in Humans

There is plenty of evidence in humans that the endocannabinoid system is involved in the regulation of the homeostasis of body composition and food intake and that it is chronically activated both in the brain and in peripheral organs after high-fat feeding and/or in obesity [168–170]. An increase of AEA and 2-AG has been observed in overweight and obese subjects [153, 171, 172]; specifically, a study on visceral adipose tissue (VAT) in lean, subcutaneous obese, and visceral obese subjects has clearly shown that 2-AG plasma concentrations were increased predominantly in abdominal obese individuals. In addition, a significant correlation between 2-AG plasma levels and visceral fat mass was found [151]. This increase of 2-AG plasma levels was in agreement with previous findings in postmenopausal women [173]. Interestingly, it has been observed that the negative relationship between circulating 2-AG and insulin sensitivity was independent of any effect on fat mass, which may imply additional effects of the endocannabinoid system on peripheral tissues. Circulating 2-AG and AEA were also found increased in type 2 diabetes patients [174]. Higher 2-AG, but not AEA, plasma content has been described in VAT of obese patients [174], probably for the increased supply of endocannabinoid precursors and/or increased activity of enzymes involved in endocannabinoid synthesis [168] and/or a decreased endocannabinoid degradation. In fact a significant downregulation of FAAH gene expression in adipose tissue of obese compared with lean individuals was found [151]. However, it cannot be ruled out that a contribution of the 2-AG circulating levels may derive from TAG hydrolysis by hormone sensitive lipase in visceral adipose tissue, which is particularly active in obese individuals.

Activation of central CB1 receptors promotes food intake and thereby weight gain [175–177] by increasing de novo lipogenesis by upregulating the lipogenic transcription factor SREBP-1c [178] and lipoprotein lipase activity [179]. 

The pharmacological approach to downregulate the endocannabinoid system with a cannabinoid CB1 receptor antagonist failed because of the increased incidence of depression and anxiety in obese subjects [180, 181]. Therefore a nutritional approach may represent an effective and devoid of adverse effects way to modulate the endocannabinoid system. Another effective strategy, combining supplements like n-3 HPUFA, with pharmaceutical treatment with thiazolidinediones, has been demonstrated to be very effective for obesity-associated disorders [182]. 

In humans, only few studies have been carried out on the potential benefits of a downregulation of the endocannabinoid system by n-3 HPUFAs PLs form on obesity-associated diseases. A recent study investigated the effect of relatively low doses of n-3 HPUFA, taken as 2 g/d of either KO or FO, 309 mg/d of EPA/DHA 2 : 1, and 390 mg/d of EPA/DHA 1 : 1, respectively, or olive oil for four weeks, on plasma endocannabinoids in overweight and obese subjects [183]. The results confirmed data in the literature [153, 171, 172], showing plasma AEA and 2-AG levels significantly higher in obese and overweight subjects and showed that KO, but not FO or olive oil, was able to significantly decrease 2-AG, although only in obese subjects. Even if the effects of the two n-3 HPUFA treatments in fatty acid PL plasma profile were similar, KO was more powerful than FO in inducing endocannabinoid changes. Interestingly, the decrease of 2-AG was correlated to the plasma PL n-6/n-3 HPUFA ratio, caused, probably, by the replacement of 2-AG precursor, ARA, with n-3 HPUFAs, as described in obese Zucker rats [49]. However, neither KO nor FO was able to affect metabolic syndrome parameters probably because of the short-period treatment. In fact, in a very recent study [41], where hypertriglyceridemic mildly obese subjects were treated up to 24 weeks with 4 g/d with krill powder containing about 400 mg of EPA + DHA, a significant decrease of triglyceridemia was associated with a steeply reduction of circulating levels of AEA. In addition, a significant decrease of the waist/hip ratio and visceral fat/skeletal muscle mass ratio was found.

Thus, it seems that dietary EPA and DHA in the PLs form may reestablish the physiological endocannabinoid tone at CB1 receptors, upregulated with visceral obesity, dyslipidaemia, insulin resistance, and atherogenic inflammation [180], through a decrease of the n-6/n-3 HPUFA ratio and thereby reduction of the endocannabinoid precursors.

## 10. Conclusions

Research on n-3 PUFAs has made important progress in different areas. Nevertheless, there are still some issues, such as efficacy according to the dietary form and putative mechanisms of action that should be better characterized. It is clearly emerging that dietary PL-bound EPA and DHA affect endocannabinoid biosynthesis at much lower doses than EPA and DHA in TAG form, probably because of the higher incorporation into tissue PLs, and positively modify several parameters of the metabolic syndrome.

EPA and DHA esterified to PLs are one of the major n-3 HPUFA dietary forms in our diet. We therefore have been exposed to this form throughout our evolution maximizing the ability to fully exploit the EPA and DHA nutritional properties. 

Future studies should address whether the peculiar property of dietary EPA and DHA bound to PLs to modulate the endocannabinoid system is also effective in ameliorating other pathological conditions where dietary EPA and DHA seem to exert beneficial activities such as cancer and psychiatric disorders. 
